# Supplementary material for: Design and Synthesis of Free-Radical/Cationic Photosensitive Resin Applied for 3D Printer with Liquid Crystal Display (LCD) Irradiation
Source: Polymers (Basel). 2020 Jun 15;12(6):1346. doi: 10.3390/polym12061346 (PMC7361968; doi:10.3390/polym12061346)
Supplement: Supplementary file 1 [file polymers-12-01346-s001.docx]

Supporting information


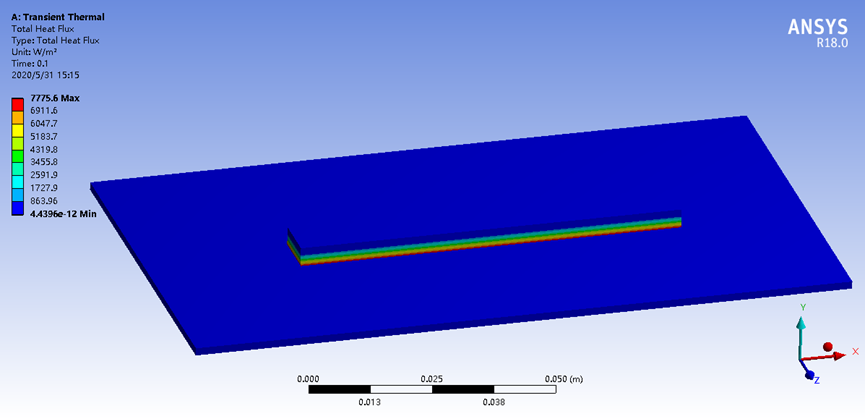


Figure S1 Heat flux maps of the spline at 80 °C


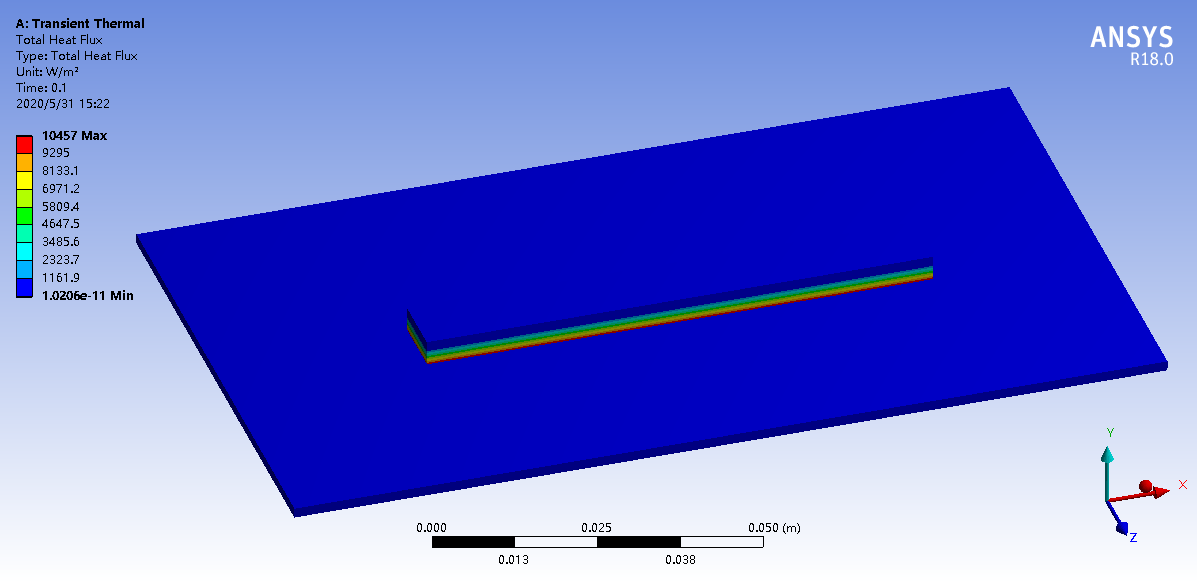


Figure S2 Heat flux maps of the spline at 100 °C


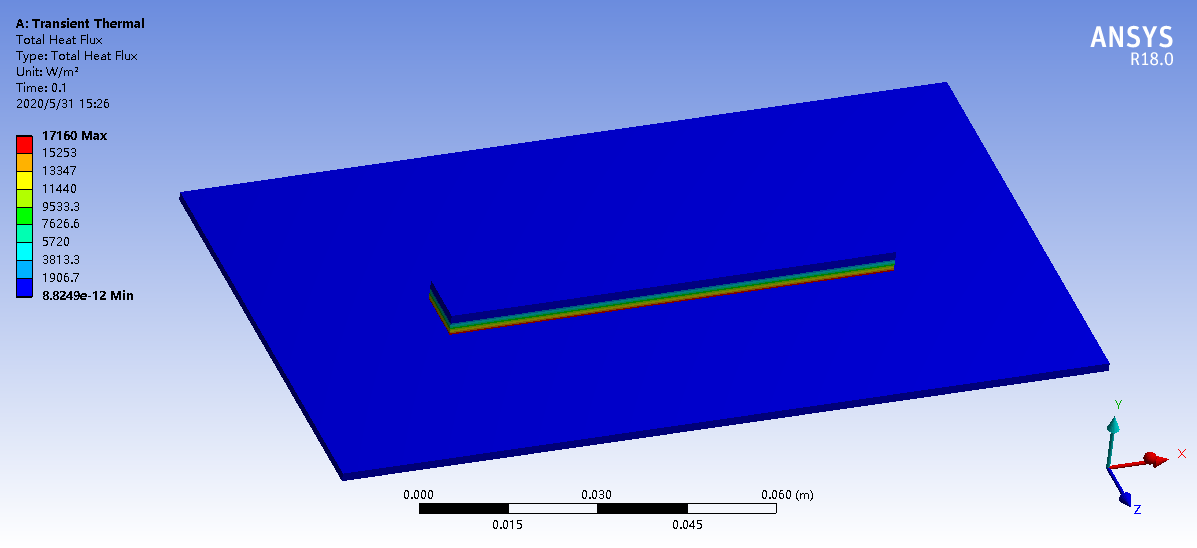


Figure S3 Heat flux maps of the spline at 150 °C


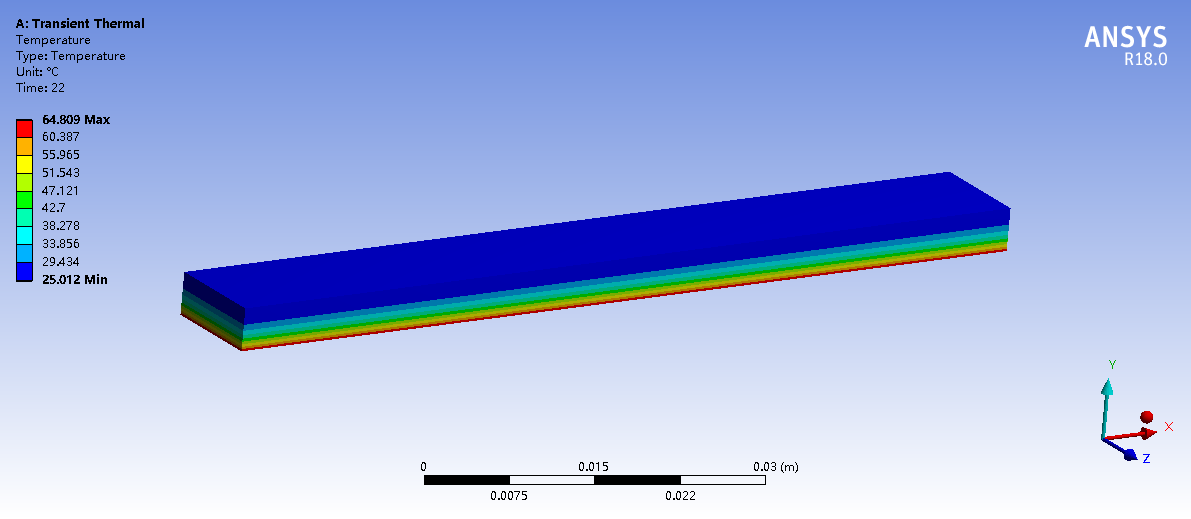


Figure S4 Temperature map of the spline with heat treatment at 80 °C for 22 s


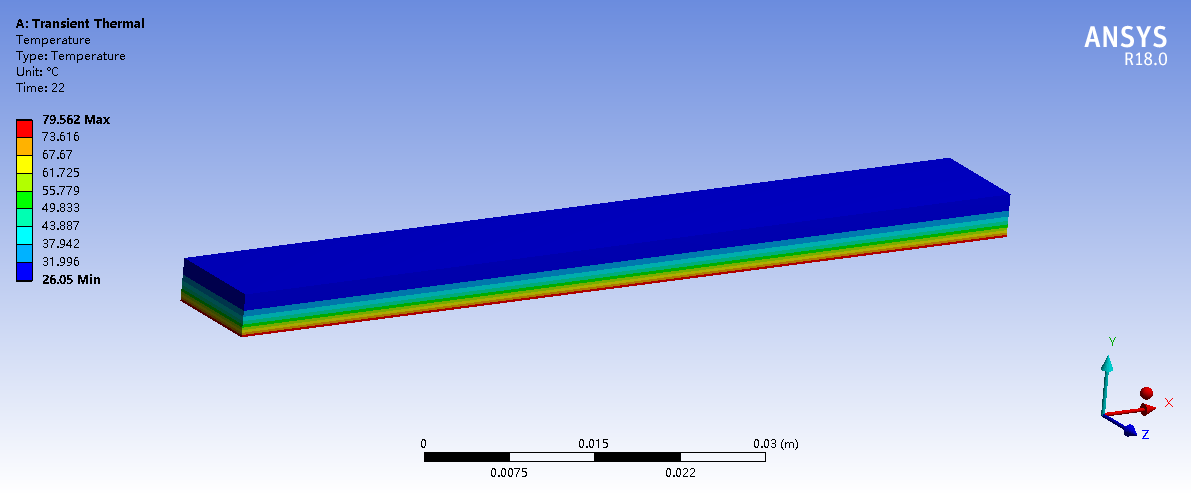


Figure S5 Temperature map of the spline with heat treatment at 100 °C for 22 s


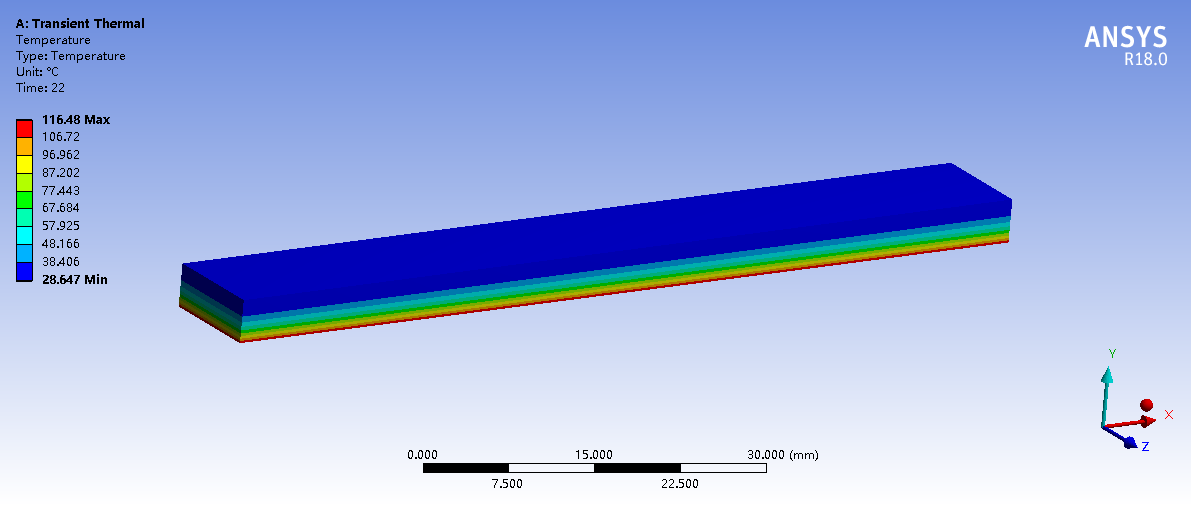


Figure S6 Temperature map of the spline with heat treatment at 150 °C for 22 s


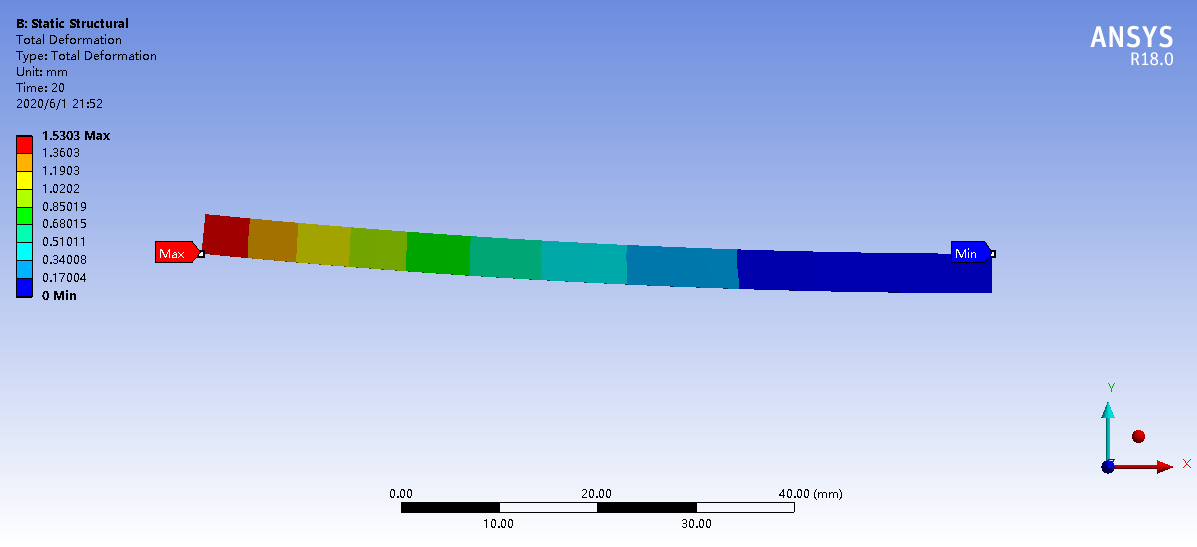


Figure S7 Maximum thermal deformation map of the spline on Y-axis at 80 °C


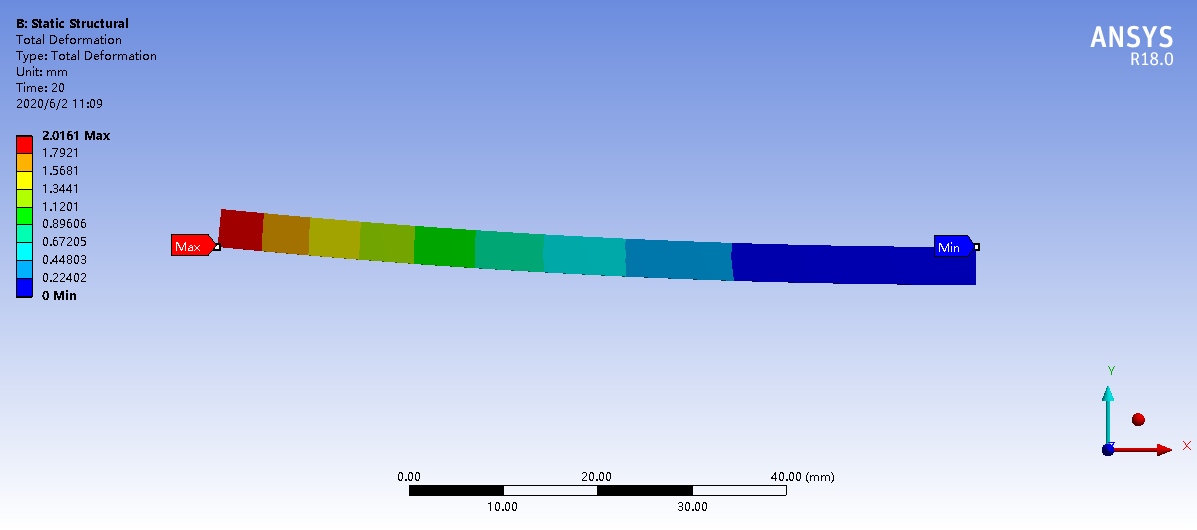


Figure S8 Maximum thermal deformation map of the spline on Y-axis at 100 °C


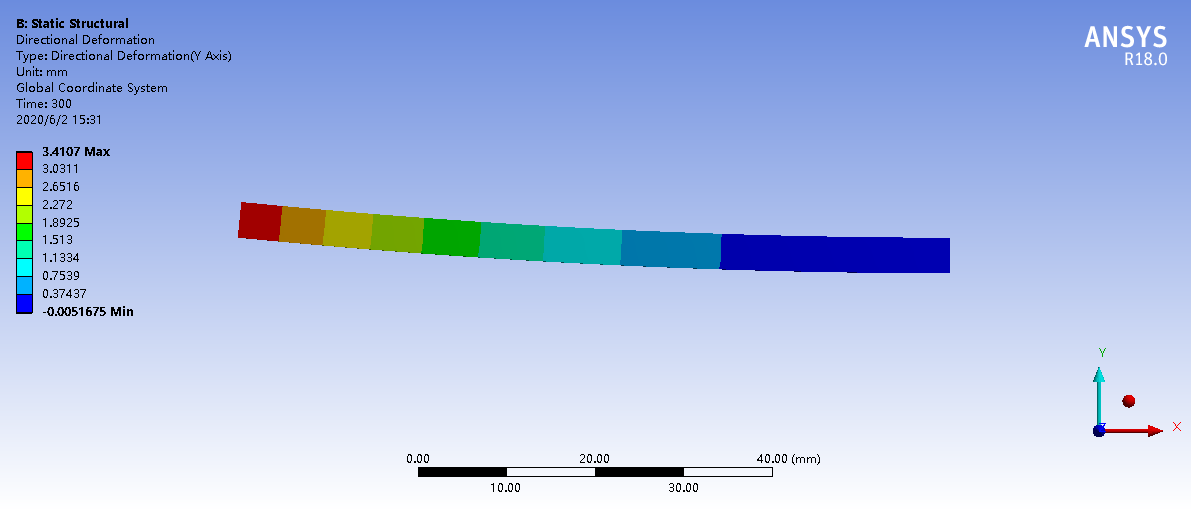


Figure S9 Maximum thermal deformation map of the spline on Y-axis at 150 °C
